# Beyond Assumptions: Racial and Delivery Method Outcomes in Pregnant Patients With Chiari Malformation

**DOI:** 10.7759/cureus.98027

**Published:** 2025-11-28

**Authors:** Chimezie Amaefuna, Rawan Elkomi, Ronak Bhatia, Amari Eubanks, Madison L Burnard, Sierra Lyles, Miriam Michael

**Affiliations:** 1 Neurology, Howard University College of Medicine, Washington, D.C., USA; 2 Obstetrics and Gynecology, Howard University Hospital, Washington, D.C., USA; 3 Obstetrics and Gynecology, Howard University College of Medicine, Washington, D.C., USA; 4 Internal Medicine, Howard University Hospital, Washington, D.C., USA

**Keywords:** chiari malformation, eclampsia, gestational hypertension, maternal outcomes, preeclampsia

## Abstract

Background: Neurological disorders can significantly influence obstetric management and outcomes, requiring careful multidisciplinary coordination. Arnold-Chiari (AC) malformation presents unique considerations during pregnancy and delivery due to its neurological implications. While cesarean delivery is often considered for safety, limited data exist regarding maternal outcomes across different racial groups and delivery methods in AC malformation patients.

Objective: This study aimed to assess maternal and obstetric outcomes by delivery method (vaginal vs. cesarean) in Black and White pregnant patients diagnosed with Chiari malformation.

Methods: A retrospective cohort study was conducted using TriNetX, an electronic health record-based research network. Separate cohorts of Black and White pregnant patients with Chiari malformation (ICD-10 Q07.0) were constructed. Patients were stratified by delivery method using the Current Procedural Terminology (CPT) codes for vaginal and cesarean delivery. Maternal outcomes were compared using ICD-10 codes for gestational hypertension (O13), preeclampsia (O14), eclampsia (O15), perineal laceration (O70), and other labor-related complications (O60-O77).

Results: Among White patients (n=1522), vaginal delivery was associated with a significantly lower risk of preeclampsia compared to cesarean delivery (4% vs. 7.1%; p=0.02). This association was not statistically significant in Black patients (4.1% vs. 5.7%; p=0.246). Although eclampsia was higher in vaginal deliveries for both races, it did not reach statistical significance. Perineal lacerations were significantly more common in vaginal deliveries among Black patients (12.7% vs. 2%; p<0.001). No significant racial differences were found in rates of headache, fetal heart abnormalities, or hypertensive disorders.

Conclusion: White patients with AC malformation had a significantly increased risk of preeclampsia following cesarean delivery, suggesting a potential benefit of vaginal delivery when feasible. Racial differences in maternal outcomes were limited but suggest potential disparities in delivery-related complications such as perineal trauma. Further studies adjusting for socioeconomic and clinical covariates are warranted.

## Introduction

Chiari malformation encompasses a group of structural anomalies at the craniovertebral junction, in which cerebellar tissue extends through the foramen magnum into the spinal canal, disrupting normal cerebrospinal fluid (CSF) dynamics [1]. Chiari malformation type I (CM-I), the most prevalent form in adolescents and adults, is characterized by the caudal displacement of the cerebellar tonsils without involvement of the brainstem [2]. In contrast, Chiari malformation type II (CM-II), historically referred to as Arnold-Chiari (AC) malformation, involves herniation of both the cerebellum and brainstem and is typically associated with myelomeningocele, a severe form of spina bifida [3].

Pregnancy in individuals with CM-I, a condition estimated to occur in approximately one of 1000 live births, presents unique clinical considerations [2]. The physiologic changes of pregnancy, particularly labor-related increases in intracranial pressure (ICP) during Valsalva maneuvers, raise theoretical concerns about symptom exacerbation, neurologic deterioration, or even herniation [4]. Historically, these concerns have prompted recommendations for cesarean delivery to mitigate risk [5]. However, recent literature suggests that both vaginal and cesarean deliveries can be safely performed in patients with CM-I when managed appropriately, including the use of neuraxial anesthesia, without documented cases of brainstem herniation [5].

These neurologic concerns must also be considered within the broader context of obstetric health disparities. Black patients in the United States face disproportionately higher rates of cesarean delivery, hypertensive disorders of pregnancy, and maternal morbidity and mortality when compared to their White counterparts [6,7]. These disparities are likely to be further compounded in the setting of complex neurological conditions, such as CM-I, where evidence-based guidelines are limited and care pathways may vary significantly.

Despite growing recognition of both Chiari malformation and longstanding racial inequities in maternal health outcomes, there remains a paucity of data examining how delivery method and race intersect to influence outcomes in this population. This study seeks to fill that gap by investigating maternal complications by delivery mode among Black and White patients diagnosed with Chiari malformation.

Research question

Are there differences in maternal outcomes by delivery method and race among patients with Chiari malformation?

Hypothesis

Maternal complications differ by delivery method, with no significant racial disparity after stratification.

## Materials and methods

Study design and data source

We conducted a retrospective cohort study using the TriNetX Research Network (accessed June 24, 2025, 18:25:43 UTC), a federated electronic health record (EHR) platform aggregating de-identified clinical data from academic medical centers, health systems, and specialty care providers across the United States. The network includes longitudinal records dating back to 2009. The ICD-10-CM code Q07.0 ("Arnold-Chiari malformation") was used to identify cases, as the system does not distinguish between Chiari subtypes. While the code encompasses both type I and type II malformations, type I is more commonly observed in isolation without neural tube defects.

Inclusion and exclusion criteria

Patients were eligible for inclusion if they met the following criteria: (1) female sex, (2) a documented diagnosis of AC malformation (ICD-10-CM: Q07.0), and (3) receipt of obstetric care associated with the diagnosis. Two delivery cohorts were defined: vaginal delivery only, with or without episiotomy or forceps (CPT: 1014217), and cesarean delivery (CPT: 1008991). The index event was defined as the first qualifying delivery event occurring within the past 20 years from the date of analysis. Only patients with data available in participating institutions of the US Collaborative Network were included.

Patients were excluded if they met any of the following criteria: (1) index delivery event occurring ≥20 years prior to analysis and (2) delivery procedures not meeting the definition of vaginal-only or cesarean-only. In the White patient analysis, 12 patients in the vaginal delivery group and 39 patients in the cesarean group were excluded due to index events occurring beyond 20 years. In the Black patient analysis, no patients were excluded on this basis. Race itself was not an exclusion criterion; however, subgroup analyses were stratified by race, and propensity score matching was applied in the Black patient analysis to balance baseline characteristics between cohorts.

Cohort selection

Pregnant patients with a recorded diagnosis of Chiari malformation were identified using ICD-10 code Q07.0 from January 1, 2009, through the most recent data available on June 24, 2025. Patients were stratified by self-reported race (Black or White). Delivery method was classified using the Current Procedural Terminology (CPT) codes for vaginal and cesarean delivery.

Matching and covariate adjustment

Separate propensity score matching (1:1) was performed within each racial group to reduce confounding, matching patients by age and ethnicity to create comparable vaginal and cesarean delivery cohorts.

Outcome measures

Primary outcomes were maternal complications during the delivery hospitalization, including gestational hypertension, preeclampsia, eclampsia, perineal laceration, and other obstetric complications (O60-O77).

Outcome definitions

Maternal outcomes were identified using ICD-10 codes for gestational hypertension (O13), preeclampsia (O14), eclampsia (O15), perineal laceration (O70), and other labor- and delivery-related complications (O60-O77).

Statistical analysis

Descriptive statistics summarized cohort characteristics. Comparative analyses assessed differences in outcomes between delivery methods within each racial group. Risk differences, risk ratios, and 95% confidence intervals (CIs) were calculated, with statistical significance set at a two-sided p-value of <0.05. Analyses were conducted within TriNetX's Health Insurance Portability and Accountability Act (HIPAA)-compliant analytics environment, which enables federated analysis across participating institutions.

## Results

After 1:1 propensity score matching by age and ethnicity, a total of 1347 White and 731 Black female patients with AC malformation were included in the analysis. Each racial group was divided into two cohorts based on mode of delivery, vaginal versus cesarean section, and assessed for differences in maternal complications.

Gestational hypertension without significant proteinuria

The incidence of gestational hypertension without significant proteinuria did not significantly differ by mode of delivery in either racial group. Among White patients, 12 of 342 individuals (3.5%) who underwent vaginal delivery were diagnosed with gestational hypertension, compared to 27 of 706 individuals (3.8%) in the cesarean delivery group. The risk difference between these groups was −0.003 (95% CI: −0.027 to 0.021), with a risk ratio of 0.917 (95% CI: 0.471 to 1.789) and an odds ratio of 0.914 (95% CI: 0.457 to 1.828) (p=0.800). Among Black patients, gestational hypertension was observed in 20 of 513 individuals (3.9%) in both the vaginal and cesarean delivery groups. The corresponding risk difference was 0.000 (95% CI: −0.024 to 0.024), the risk ratio was 1.000 (95% CI: 0.545 to 1.836), and the odds ratio was 1.000 (95% CI: 0.531 to 1.882), with a p-value of 1.000.

Preeclampsia

**Figure 1:**
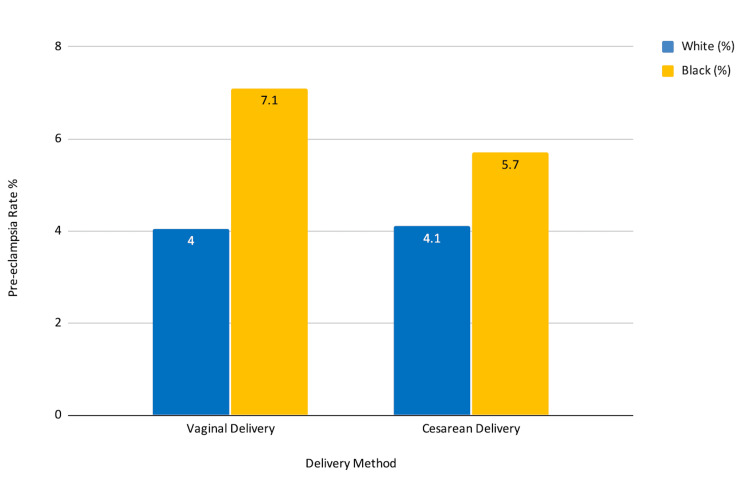
Preeclampsia rate by delivery method and race This bar chart displays the proportion of patients diagnosed with preeclampsia, stratified by delivery method (vaginal vs. cesarean) and race (White vs. Black), in a retrospective cohort analysis of pregnant patients with Chiari malformation using the TriNetX Research Network. The cohort included 519 White patients with vaginal delivery, 1003 White patients with cesarean delivery, 513 Black patients with vaginal delivery, and 513 Black patients with cesarean delivery. Preeclampsia diagnoses were identified using the ICD-10 code O14. Rates are expressed as percentages of each subgroup. Statistical analysis showed that, among White patients, preeclampsia incidence was significantly lower in vaginal deliveries (4%) compared with cesarean deliveries (7.1%; p=0.019). Among Black patients, preeclampsia rates were 4.1% for vaginal and 5.7% for cesarean deliveries, a difference that was not statistically significant (p=0.246).

Among White patients, preeclampsia occurred in 21 of 519 individuals (4%) who delivered vaginally and 71 of 1,003 individuals (7.1%) who underwent cesarean delivery (Figure 1). While preeclampsia was more frequently documented in the cesarean group, this difference likely reflects clinical selection for cesarean delivery after the diagnosis of preeclampsia, rather than a protective effect of vaginal delivery.

Among Black patients, preeclampsia occurred in 21 of 513 individuals (4.1%) who delivered vaginally and 29 of 513 individuals (5.7%) who underwent cesarean delivery. The risk difference was −0.016 (95% CI: −0.042 to 0.011), with a risk ratio of 0.724 (95% CI: 0.419 to 1.253) and an odds ratio of 0.712 (95% CI: 0.401 to 1.267). This difference was not statistically significant (p=0.246). 

Eclampsia

**Figure 2:**
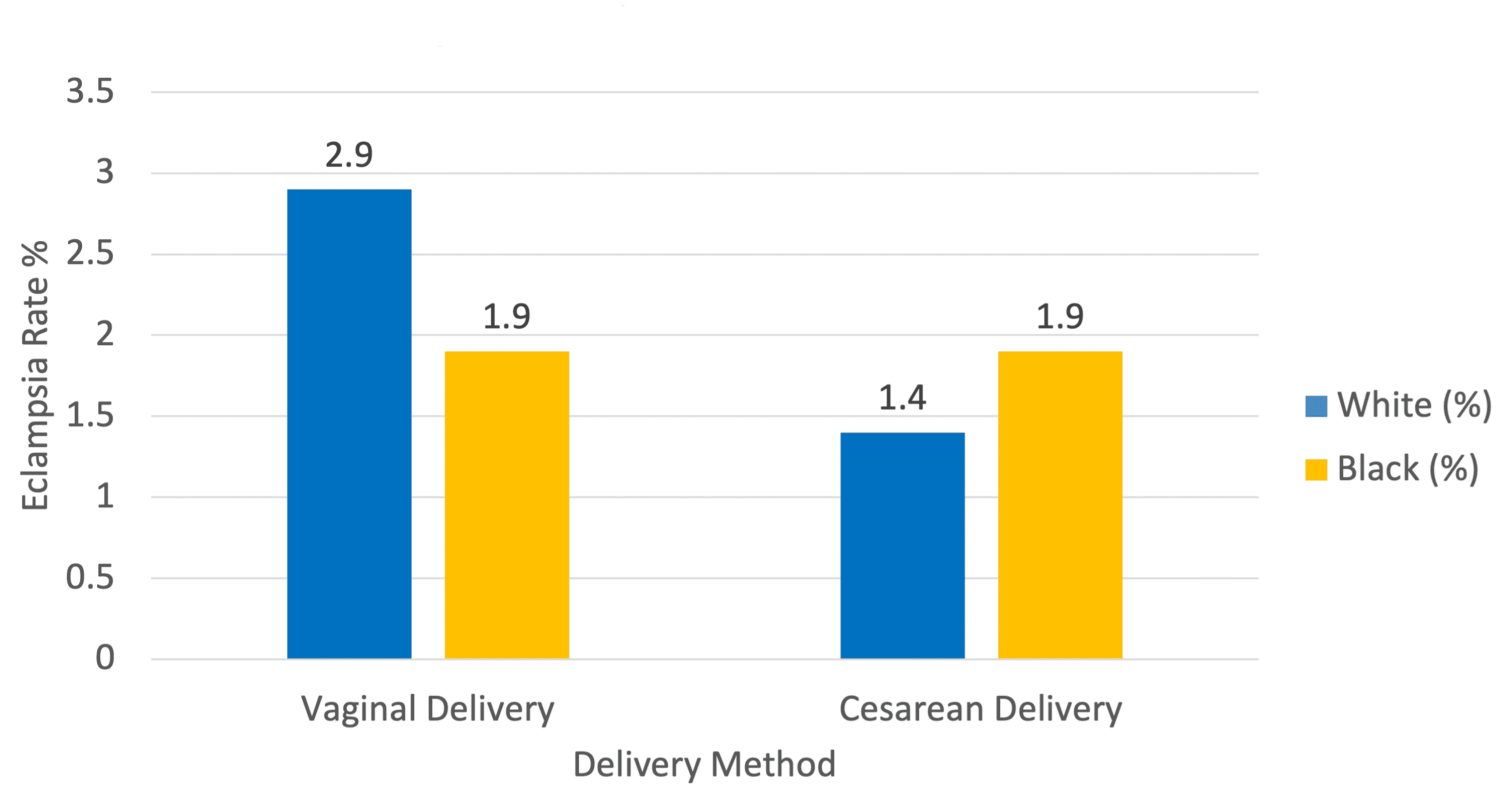
Eclampsia rate by delivery method and race This bar chart displays the proportion of patients diagnosed with eclampsia, stratified by delivery method (vaginal vs. cesarean) and self-reported race (White vs. Black), in a retrospective cohort study of pregnant patients with Chiari malformation using the TriNetX Global Research Network. The cohort included 519 White patients with vaginal delivery, 1003 White patients with cesarean delivery, 513 Black patients with vaginal delivery, and 513 Black patients with cesarean delivery. Eclampsia diagnoses were identified using the ICD-10 code O15. Rates are expressed as percentages of each subgroup. Among vaginal deliveries, the eclampsia rate was higher in White patients (2.9%) compared to Black patients (1.9%). In contrast, among cesarean deliveries, Black patients had a slightly higher rate (1.9%) than White patients (1.4%). Statistical analysis did not demonstrate a significant difference in eclampsia rates between delivery methods within either racial group.

The incidence of eclampsia was low and did not significantly differ by delivery method in either racial group. Among White patients, eclampsia was observed in 10 of 342 individuals (2.9%) who underwent vaginal delivery and in 10 of 706 individuals (1.4%) who had a cesarean section (Figure 2). This difference was not statistically significant, and contrary to prior drafts, eclampsia was not exclusive to vaginal deliveries. The risk difference between groups was 0.015 (95% CI: −0.005 to 0.035), with a risk ratio of 2.064 (95% CI: 0.867 to 4.912) and an odds ratio of 2.096 (95% CI: 0.864 to 5.086), yielding a p-value of 0.094. In the Black patient cohort, 10 of 513 individuals (1.9%) were diagnosed with eclampsia in both the vaginal and cesarean groups, resulting in a risk difference of 0.000 (95% CI: −0.017 to 0.017), a risk ratio of 1.000 (95% CI: 0.420 to 2.382), and an odds ratio of 1.000 (95% CI: 0.413 to 2.423) with a p-value of 1.000. These findings indicate that the mode of delivery was not significantly associated with the risk of eclampsia among either White or Black patients.

Perineal laceration during delivery

**Figure 3:**
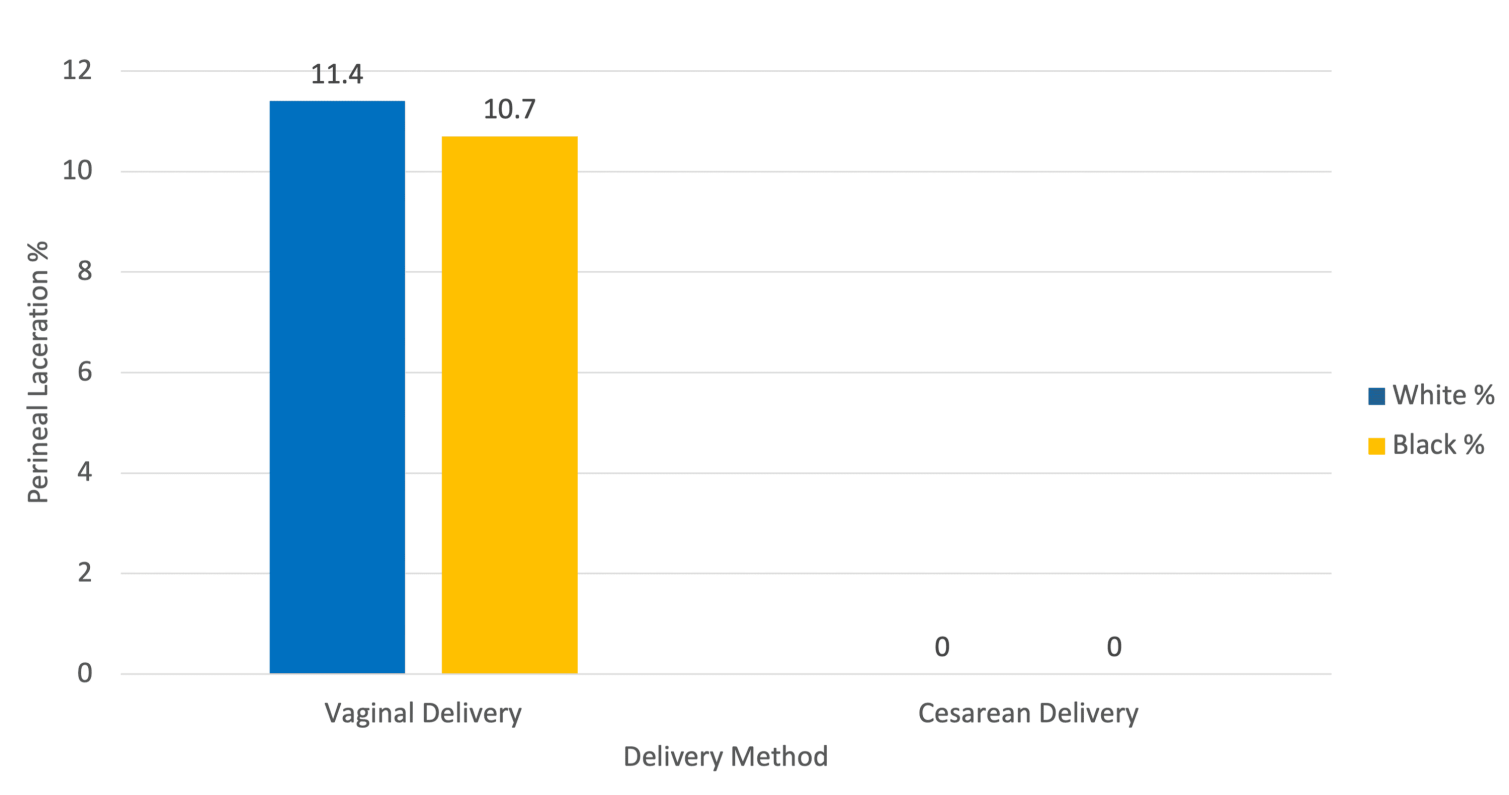
Perineal laceration rate by delivery method and race This figure illustrates the percentage of perineal lacerations among pregnant patients with Chiari malformation, stratified by delivery method and race. The data are drawn from a retrospective cohort study using the TriNetX Global Research Network. Lacerations were identified using the ICD-10 code O70. Among those who delivered vaginally, 11.4% of White patients and 10.7% of Black patients experienced a perineal laceration. No lacerations were recorded for either group following cesarean delivery, as expected due to surgical avoidance of the perineum.

Perineal lacerations occurred exclusively in vaginal deliveries, which is an expected mode-specific finding rather than a Chiari-related complication [8]. Among White patients, 39 of 342 (11.4%) who delivered vaginally experienced perineal laceration, while none (0 of 706) in the cesarean group did (Figure 3). This resulted in a risk difference of 0.114 (95% CI: 0.082 to 0.147), which was statistically significant (p<0.001). Among Black patients, the incidence was similarly elevated in vaginal deliveries, with 55 of 513 (10.7%) experiencing perineal laceration compared to 0 of 834 in the cesarean group, yielding a risk difference of 0.107 (95% CI: 0.080 to 0.134) (p<0.001).

Labor and delivery complications

Other labor and delivery complications (ICD-10 codes O60-O77) occurred in 19 of 427 (4.4%) vaginal deliveries and 22 of 877 (2.5%) cesarean deliveries. Although this reflects a higher incidence in the vaginal delivery group, the difference did not meet statistical significance (p=0.059).

Abnormal fetal heart rate

Abnormal fetal heart rate and rhythm (ICD-10: O76) were reported in 10 of 427 vaginal deliveries (2.3%) and 10 of 877 cesarean deliveries (1.1%). The difference approached statistical significance but did not meet conventional thresholds (p=0.098).

Headache

Postpartum headache rates were comparable across delivery methods and racial groups. Among White patients, headache was documented in 18 of 342 vaginal deliveries (5.3%) and 41 of 706 cesarean deliveries (5.8%) (p=0.726). Among Black patients, the rates were similarly consistent: 27 of 513 vaginal deliveries (5.3%) and 45 of 834 cesarean deliveries (5.4%) (p=0.925).

Hemorrhages: intrapartum and postpartum

Intrapartum Hemorrhage

The incidence of intrapartum hemorrhage (ICD-10: O67) significantly differed by mode of delivery. Among those with vaginal delivery, 10 of 514 patients (1.9%) experienced intrapartum hemorrhage. No patients (0/977) in the cesarean delivery group experienced this complication. The calculated risk difference was 0.019 (95% CI: 0.008 to 0.031), with a p-value of <0.001, indicating a statistically significant difference. This suggests a higher risk of intrapartum hemorrhage among vaginally delivering Chiari patients. Risk and odds ratios could not be computed due to a zero event count in the cesarean group.

Postpartum Hemorrhage

Postpartum hemorrhage (ICD-10: O72) rates were comparable across both racial groups and modes of delivery, with no statistically significant differences observed. Among White patients, 15 of 342 individuals (4.4%) who delivered vaginally experienced postpartum hemorrhage, compared to 28 of 706 (4%) who delivered via cesarean section. The risk difference was 0.004 (95% CI: −0.019 to 0.027), with a risk ratio of 1.097 (95% CI: 0.589 to 2.045) and an odds ratio of 1.095 (95% CI: 0.570 to 2.104) (p=0.781). Among Black patients, postpartum hemorrhage occurred in 19 of 513 individuals (3.7%) who delivered vaginally and 36 of 834 (4.3%) who delivered via cesarean section, yielding a risk difference of −0.006 (95% CI: −0.026 to 0.015), a risk ratio of 0.873 (95% CI: 0.504 to 1.513), and an odds ratio of 0.863 (95% CI: 0.489 to 1.521) (p=0.613). These findings suggest that postpartum hemorrhage is not significantly associated with delivery mode or race among patients with Chiari malformation.

## Discussion

This study represents one of the most comprehensive racially stratified investigations of maternal outcomes in patients with Chiari malformation, evaluating delivery-associated complications across vaginal and cesarean modes. Our findings suggest that, in most cases, the mode of delivery is not significantly associated with increased risk of hypertensive complications, including gestational hypertension, preeclampsia, or eclampsia. This reinforces the safety of both vaginal and cesarean delivery in appropriately selected patients, supporting prior case reports and small series that have advocated for individualized delivery planning with careful anesthetic management [9,10]. 

With regard to preeclampsia, because outcomes were assessed during the delivery hospitalization, the temporal sequence between preeclampsia diagnosis and delivery mode could not be established. It is therefore unclear whether preeclampsia developed prior to the decision for cesarean delivery or was recorded as part of the delivery encounter.

However, two complications, perineal laceration and intrapartum hemorrhage, were observed exclusively among vaginal deliveries and were statistically significant in both racial groups. While these outcomes are expected with vaginal delivery, they may carry additional implications in the Chiari population, where alterations in CSF dynamics and neural sensitivity may amplify pain, recovery time, or complications. Previous studies have noted that Chiari I patients may be at increased risk for pelvic floor dysfunction and pain syndromes, potentially heightening the impact of obstetric trauma [11].

Intrapartum hemorrhage, though generally uncommon, showed a statistically significant increase in vaginal deliveries and warrants special consideration in the Chiari population. Dysregulation of autonomic tone, impaired venous return, or reduced baroreceptor sensitivity, features potentially associated with Chiari malformation, could influence hemodynamic stability during labor and delivery [12]. Further research is needed to determine whether these neurologic vulnerabilities translate into elevated hemorrhagic risk and whether enhanced monitoring or preventive strategies could mitigate complications in this subset of patients [13].

Additionally, the absence of significant differences in hypertensive complications across delivery methods may reflect the importance of modern anesthetic techniques in reducing these complications. Neuraxial anesthesia, when carefully titrated, can reduce intrapartum stress and ICP fluctuations without compromising uteroplacental perfusion. These benefits may be especially relevant in Chiari patients where elevated ICP is a concern [14]. These results echo prior literature suggesting that with adequate planning, vaginal delivery is a feasible and often safe option [15].

Our racially inclusive design also reinforces the importance of addressing disparities in obstetric care. Black patients in the United States face persistently higher cesarean rates and worse maternal outcomes, often independent of clinical indication [16]. Although our study was not powered to detect nuanced racial disparities in outcomes, the inclusion of both Black and White patients provides foundational data for future studies. It highlights the urgent need for equitable, standardized care pathways in neurologic obstetrics [17,18].

Limitations

This study has similar limitations inherent to any retrospective study and reliance on EHRs. Despite propensity score matching, residual confounding from unmeasured variables, such as socioeconomic status, precise cesarean indications, body mass index (BMI), and lifestyle factors, may remain. Outcome ascertainment depended on diagnostic and procedure codes, introducing potential misclassification bias. Additionally, the TriNetX platform lacks granular obstetric data such as gestational age, parity, fetal presentation, and estimated blood loss, limiting adjustment for clinical nuance. Race and ethnicity data, captured through EHR documentation, may be incomplete or inconsistently reported and do not fully encompass social determinants contributing to observed disparities. The analysis also cannot delineate the timing or severity of complications nor assess long-term maternal or neonatal outcomes. Although TriNetX provides comprehensive EHR data, certain key obstetric and demographic covariates were unavailable for this analysis. Specifically, variables such as BMI, parity, gestational age at delivery, prior cesarean history, induction or augmentation of labor, anesthetic type, socioeconomic status, and hospital region or type were not consistently captured across participating institutions. Finally, because the data originate from a US-based consortium of predominantly large health systems, the findings may not be generalizable to other countries or to patients receiving care in smaller or non-participating hospitals.

Implications

These findings have clinical implications for delivery planning in patients with Chiari malformation. Although cesarean delivery is often favored due to theoretical concerns about ICP elevation, our results support the broader literature indicating that vaginal delivery may be appropriate in many cases. However, given the exclusive occurrence of eclampsia and perineal laceration in the vaginal group, individualized delivery planning, especially for patients with a history of hypertensive disorders, is essential.

## Conclusions

This study evaluated maternal outcomes by delivery method among Black and White patients with Chiari malformation, with the goal of informing obstetric decision-making in a neurologically complex population. While most outcomes did not differ significantly between delivery methods, certain complications, such as eclampsia and perineal laceration, occurred exclusively among vaginal deliveries, reflecting expected, mode-specific findings rather than Chiari-specific risks.

These results, derived from a relatively large and racially inclusive cohort, emphasize the importance of individualized delivery planning rather than a one-size-fits-all approach. They also support the judicious, evidence-informed use of vaginal delivery when clinically appropriate, challenging the long-standing assumption that cesarean delivery is inherently safer for patients with Chiari malformation.

Future research should incorporate imaging data, longitudinal follow-up, patient-reported outcomes, and provider decision-making factors to clarify causal pathways and long-term implications. Expanding investigations into racial and socioeconomic disparities in neurologically complex pregnancies will be essential to advancing equitable, evidence-based maternal care for individuals with Chiari malformation.
